# Fournier’s gangrene: its management remains a challenge

**DOI:** 10.11604/pamj.2021.38.23.25863

**Published:** 2021-01-12

**Authors:** Faiez Boughanmi, Farouk Ennaceur, Ibtissem Korbi, Amina Chaka, Faouzi Noomen, Khadija Zouari

**Affiliations:** 1 Department of General and Digestive Surgery, University Hospital Fattouma Bourguiba, University of Monastir, Monastir, Tunisia

**Keywords:** Fournier’s gangrene, wound healing techniques, restorative surgery, vacuum assisted closure, therapy, hyperbaric oxygenation

## Abstract

Fournier's gangrene (FG) is a rapidly progressive necrotizing bacterial dermo-hypodermitis of the perineum and external genitalia. It represents a real medical and surgical emergency requiring multidisciplinary care. Our study was based on the retrospective analysis of 18 cases of FG, collected in the Department of General and Visceral Surgery of Fattouma Bourguiba University Hospital in Monastir over an 18-year period extending from January 2000 to December 2018. Our series included 18 cases of FG collected over an 18-year period, an annual incidence of one case per year. The average age of our patients was 58 years (36 to 77). The male prevalence was clear. Diabetes and old age were found to be the major risk factors. The treatment was based on an aggressive surgical debridement remains to be the cornerstone of therapy and is commonly preceded by patient preparation for the surgical act by perioperative resuscitation and broad-spectrum antibiotic therapy, possibly accompanied by hyperbaric oxygen therapy (HBOT). The vaccum assisted closure (VAC) therapy is also used, which is a non-invasive system that promotes open wound healing. Healing techniques can be once the septic risk is controlled. Dressings topical treatments, such as fatty substances or calcium alginate, in addition to skin grafts, musculo-neurotic or musculo-cutaneous cover flaps can be used. During the follow-up period, no reccurrence occurred in 14 out of the 18 cases (2 patients were lost to follow-up and 2 patients died). A colostomy was closed in 10 out of 11 cases with simple follow-ups. Restorative surgery (partial thickness skin graft) at the perineal level was performed in only one case. Despite the better understanding of its etiopathogenesis, the advent of targeted antibiotic therapy, the establishment of a better codification of surgical procedures, the contribution of hyperbaric oxygenation and reconstruction techniques, mortality rates are still high and FG remains a real health threat, thus constituting a real medical and surgical emergency.

## Introduction

Fournier's gangrene (FG) is a rapidly progressive necrotizing bacterial dermo-hypodermitis of the perineum and external genitalia. It is secondary to a polymicrobial infection by aerobic and anaerobic bacteria having a synergistic action [1, 2]. It represents a real medical and surgical emergency requiring multidisciplinary care. The etiology is identified in 95% of cases [2]. The source of the infection is either cutaneous, urogenital or colorectal [2-4]. Favoring factors, such as age, diabetes and immunosuppression, are often present [5-7]. Clinical features are fulminant (fever, prostration, edematous erythema of the scrotum, palpation of typical scrotal crepitation). To ensure patient survival, treatment is based on the restoration of the hydro-electrolyte balance with a broad-spectrum antibiotic therapy promptly followed by aggressive surgical debridement. Mortality remains high, around 40% [1], often due to delayed diagnosis and management. Patients who survive the infection will undergo reconstruction surgeries which can sometimes be associated with very marked squeal related to the extent of the fasciitis and debridement. The aim of this work is to stress the seriousness of this pathology and the importance of a diagnosis as well as a rapid and adequate treatment to improve its prognosis.

## Methods

Our study was based on the retrospective analysis of 18 cases of FG, collected in the Department of General and Visceral Surgery of Fattouma Bourguiba University Hospital in Monastir over an 18-year period extending from January 2000 to December 2018. Our patients were recruited through the Emergency Department of Fattouma Bourguiba University Hospital in Monastir and the other emergency departments and surgical departments of the provincial hospitals in the region. Data collection was based on a review of the medical records of all patients and operative reports and reported in the form of 18 observations.

## Results

Our series included 18 cases of FG collected over an 18-year period, an annual incidence of one case per year. The average age of our patients was 58 years with extremes ranging from 36 to 77 years. The male prevalence was clear with 13 males out of the 18 patients included in this study, diabetes and old age were found to be the major risk factors. In fact, diabetes was found in the majority of the cases in our series; 12 out of 18 cases were diabetic, including three cases of first discovery and 4 out of 18 patients were aged 70 or over. The etiology was found in 14 cases out of 18, suppuration with a perianal starting point in 12 cases, urethral fistula in 1 case and acute prostatitis complication in 1 case. The average consultation time was 10 days with extremes ranging from 3 to 20 days. The predominant functional sign was a painful swelling of the perineal region. A history of diabetes was found in the majority of patients: 12 out of 18 cases, including three cases of first discovery and 6 patients had no history. Table 1 summarizes the study patients´ history.

**Table 1:** history of patients in our series

Medical history	Number of cases
Diabetes	12
Hypertension	4
Urological history	2
Anal fistula	1
Rectal cyst	1
No medical history	6

No morphological examination was carried out. The medical treatment concomitant with the surgical one was based on a hydroelectrolytic resuscitation, balance of the tares and an intravenous antibiotic therapy. The majority of our patients received dual antibiotics based on Amoxicillin-clavulanic acid (1g x 3/d) and Metronidazole (500 mg x 3/d). Other patients had a different association or were adjusted for the antibiogram (Table 2) (to the culture sensitivity of the microbial isolates).

**Table 2:** isolated germs and antibiotic therapy received by our patients

Number of cases	Isolated Germ	Ant biotherapy
1	None	Amoxicillin-clavulanic acid Métronidazol Ofloxacin
2	Pseudomonas Klebsiella	Amoxicillin-clavulanic acid Métronidazol
1	None	Imipenèm Ciprofloxacin
1	None	Céfotaxim Métronidazol Gentamycin
1	Escherichia. Coli Acinetobacter	Céfotaxim Métronidazol Gentamycin
Rest of patients	None	Amoxicillin-clavulanic acid Métronidazol

The average duration of intravenous antibiotic therapy was three to four weeks. Surgery was performed urgently (the day of admission) in all cases under general anesthesia. The surgical management of our patients was aggressive consisting of large cutaneous, subcutaneous and fascial excisions extending up to the surrounding healthy limits while obtaining clean and tonic wounds which could quickly bud after the taking of bacteriological samples. These surgical wounds were left wide open, drained by Delbet blades or by Betadine impregnated swabs and washed with isotonic saline added to Betadine and hydrogen peroxide were carried out. A temporary diversion of the fecal matter (colostomy) was performed in 11 out of the 18 patients. The average length of hospital stay was 41 days with extremes ranging from 13 to 120 days. The postoperative follow-up was simple in 13 cases. A stay exceeding 30 days was observed in 10 cases. Iterative excisions (more than two) were performed in 17 cases. A bypass colostomy was performed in 10 cases. A thromboembolic accident (ischemic stroke) was observed in one case. Decompensation of diabetes was observed in three cases. Severe sepsis was observed in one case. Septic shock was observed in two cases. Mortality was observed in two patients who had the following characteristics in common: delayed treatment, female gender, age over 65, diabetes, gangrene extension beyond the perineum and septic shock.

Regarding follow-up, surveillance included clinical follow-up. During the follow-up period, no reccurrence occurred in 14 out of the 18 cases (2 patients were lost to follow-up and 2 patients died). A colostomy was closed in 10 out of 11 cases with simple follow-ups. Restorative surgery (partial thickness skin graft) at the perineal level was performed in only one case.

## Discussion

FG is a potentially lethal form of genital, perineal and perianal necrotizing fasciitis that results from a polymicrobial infection, the source of which can be genitourinary, colorectal, cutaneous or idiopathic [1, 2]. The true incidence of the disease is not known. Currently, the incidence of severe cellulite is estimated at 0.1-0.4 per 100,000 population. In the United States, its incidence is estimated between 900 and 1,000 cases per year [2]. FG is rare. In fact, only 800 published cases, mostly in the form of isolated cases or in series have been reported [3]. This affliction is commonly seen in the 40 to 75-year age group. Men are much more affected than women which could be explained by better drainage of the perineal region in women via vaginal secretions [2, 3]. This was observed in our series where our patients had an average age of 58 years with a clear male predominance. Factors favoring the infection and the outbreak of gangrene leading to tissue damage such as ischemia or a state of immunosuppression are often found in the history.

Diabetes seems to be the most predisposing factor to the disease [5]. It is found in approximately 30% of cases in the literature [6]. Hyperglycemia has been found to be a factor affecting the adhesion, chemotaxis and bactericidal activities of phagocytes. They have also been shown to have harmful effects on cellular immunity [6-8]. Diabetes can, in fact, lead to disturbances in cellular and humoral immunity responsible for a decrease in the level of activated T lymphocytes, immunoglobulins (IgG and IgA) and complement fractions (C3 and C4) but also alterations of the bactericidal function of polynuclear neutrophils [6]. In our series, 12 out of 18 patients were diabetic. Alcoholism has been cited as a favoring factor in several series [6-8] but the association has not been clearly validated [9]. Age has also been linked to an increased tendency to develop FG as it occurs most frequently in old subjects [2, 7, 10]. Malnutrition, has also been reported as a risk factor as it weakens the host's defenses against exogenous bacterial attacks due to the acquired immunosuppression that it causes [2, 11].

Corticosteroid therapy and morbid obesity contribute to the development of a particular environment often involving a form of immunosuppression [3, 7, 12, 13]. In 1883 Fournier described this gangrene as idiopathic, brutally affecting young healthy men. Nowadays this definition has completely changed since an underlying cause can be identified in almost all cases [14]. The etiology is identified in 75 to 100% of patients [2]. The most frequent form is the so-called primitive form. In fact, all locoregional suppurations can progress to GF. Colorectal origins such as suppurations of the anal margin [2, 3, 9] and fistulization of rectal tumors [15, 16] represent around 13 to 50% [2]. Urological origins such as orchiepididymitis and acute prostatitis and complications of urethral strictures, with extravasation of urine [2, 11] account for 17% to 87% of cases [11]. Cutaneous origins including acute and chronic skin infections of the scrotum [2] and suppurative hydradenitis [2, 12] are more rarely involved.

Other rarer etiologies such as intra-abdominal infections [12] and post-operative and post traumatic FG [17] have been reported. Early diagnosis mainly depends on the clinician's alertness to symptoms and suggestive signs. However, the average time for diagnosis remains extended by an average of 6 days [2, 18, 19]. Classically, perineal gangrene progresses in four clinical phases [11]: the first phase, which lasts 24 to 48 hours, is non-specific and often insidious. It manifests as discomfort, irritability, digestive problems and / or low back pain. The second phase is the invasion phase, also of short duration, which is characterized by locoregional inflammatory manifestations. The third phase is the necrosis phase characterized by an increase in the general signs and the development of a serious infectious syndrome, which can go as far as septic shock in 50% of cases. The fourth phase is the spontaneous restoration phase: the cleansing of necrotic tissue is done in less than two weeks. The general signs gradually improve, slow healing takes place over several months. It begins with a budding of the bottom and ends with a centripetal epidemization [11, 12]. The lesions remain localized to the perineum in 50% of the cases, but they can extend in severe cases. No additional examination is necessary for the diagnosis [2].

**Figure 1:**
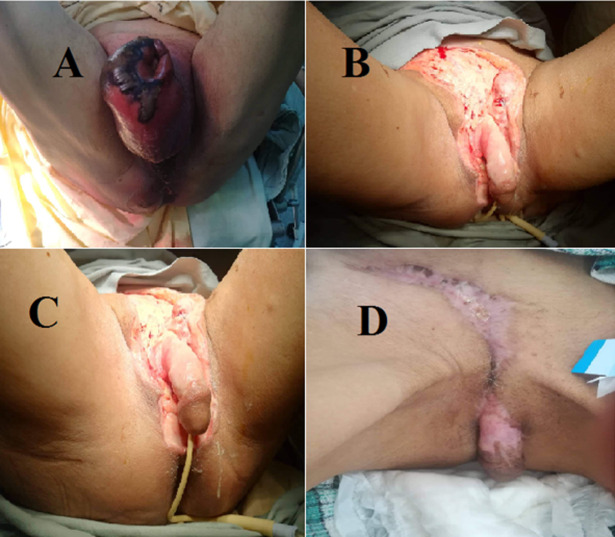
evolution of a case of FG admitted to our department: A) before performing the first debridement and orchidectomie; B) after the first debridement; C) wound evolution at day 15 post-operative; D) wound evolution a day 45 post-operative

But a biological inflammatory syndrome, hyperglycemia and hydro-electrolytic disorders, also are found in almost all cases [2, 7, 11]. Local samples with culture of purulent collections and bacteriological analysis of excised tissues must be done quickly. They allow the identification of pathogens in 80% to 95% of cases and a possible adaptation of initial antibiotic therapy [11]. The most frequently identified germs are anaerobes, the best known of which is Clostridium [20]. Other incountred bacteria include *Escherichia coli* (75.6%), streptococcus (40.5%), *Pseudomonas aeruginosa* (12%) and *proteus* [17, 20] as highlighted in Table 2. Imagery can sometimes be requested as it represents adjunct in confirming the presence of bubbles or subcutaneous gas pockets, but it should, in no case, delay therapeutic management because the diagnosis of these infections is primarily clinical .Medium term complications are primarily associated with the extended stay in an intensive care unit (thromboembolic, cardiorespiratory and cutaneous infectious). Long term complications are mainly aesthetic, functional and psychological. In addition to these, there are complications inherent to ancillary procedures: orchidectomy, penectomy, restoration of digestive continuity, reconstructions, etc. No recent study has managed to assess FG late complications [11]. Treatment must be carried out urgently. It is medical through resuscitation measures, antibiotic therapy and mainly surgical. Due to the potential severity of these infections and the progression to septic shock, it is important to hospitalize these patients in an intensive care setting and immediately initiate probabilistic antibiotic treatment, that is active against both anaerobes and negative gram bacils (BGN). Surgical treatment must be carried out as early as possible and possibly accompanied by hyperbaric oxygen therapy (HBOT). This multidisciplinary care must be implemented without delay. The treatment aims firstly to restore the hemodynamic balance by the different means of resuscitation, to excise all the necrotic tissues by surgical debridement which constitutes the cornerstone of treatment, to fight against the infectious process by a broad spectrum antibiotic therapy and finally to start the reconstruction which consists in covering the tissue defect after surgical debridement. The initial surgical treatment is a rescue therapy that must not be mutilating from the start but must lead to macroscopically healthy tissue, sometimes at the expense of a significant sacrifice of tissue. We should put loose stitches without attempting a wound tightly. It is necessary to make a bandage flat, with no fatty substance (Figure 1).

This first surgical procedure is followed by iterative dressings after 24 to 48 hours (second look) in order to monitor the progression of the disease. These are, at the beginning, performed in the operating room, under GA, daily or every other days, depending on the evolution. Healing techniques can be used during this period once the septic risk is controlled. Dressings topical treatments, such as fatty substances or calcium alginate, in addition to skin grafts, musculo-neurotic or musculo-cutaneous cover flaps can be used [7, 11, 21].

The main problem is the timing of the reconstruction. This can start as soon as the wound becomes clean. The practice of biopsies can help to know this “timing” by showing the presence of granulation tissue, which means that the level of bacteria has reached a low and satisfactory threshold. However, even if 1,000 bacteria persist, the wound may not heal. In cases of large losses of substance, surgeons often resort to the use of split-thickness skin grafts taken from the anterior side of the thigh with a thickness of 0.01 to 0.015 inches with the creation of mesh in the graft used (Figure 1) (Meshed split-thickness skingraft) [2]. The VAC (vaccum assisted closure) therapy is also used, which is a non-invasive system that promotes open wound healing through the application of negative (sub-atmospheric) pressure. The suction on the wound acts by extracting peri-lesional fluids, which reduces capillary compression and stimulates microcirculation in the early stages of inflammation [22]. Hyperbaric oxygen therapy was introduced in the treatment of gas gangrene in 1941 by Annane and Raphaël [23]. It is still discussed in the therapeutic management of FG and necrotizing cellulites in general. It is still the subject of controversy.

The principle of HBOT is that it ensures tissue over-oxygenation by association of the increase in pressure beyond 1 absolute atmosphere and the increase in partial pressure in oxygen breathed beyond a bar. HBOT causes phagocytic activation of polynuclear cells, a bactericidal effect, a bacteriostatic action, potentiation of the effects of certain antibiotics and stimulation of the healing phenomena: angiogenesis and re-epithelization [23]. In terms of prognosis, FG is a serious condition that causes mortality ranging from 20% to 50%. This mortality is all the more important when the management is initiated tardily, when the patient is immune-compromised or when there is a state of septic shock [3, 5]. Prognostic factors vary from one study to another, but the most cited factors are: female gender, age over 65, diabetes, the extension of gangrene beyond the perineum, delay in consultation, delayed management, elective colostomy and septic shock [7, 11, 24].

## Conclusion

In summary, FG is a serious infectious disease that is often diagnosed late. It mainly affects fragile patients with impaired immune defenses. Its diagnosis is primarily clinical with an alarming presentation in most cases. It represents a real medical and surgical emergency. Due to the rapidity of its progression, urgent and multidisciplinary care is vital. In the acute phase, aggressive surgical debridement remains to be the cornerstone of therapy and is commonly preceded by patient preparation for the surgical act by perioperative resuscitation and broad-spectrum antibiotic therapy. The leading factors associated with high morbidity and mortality are as follows: advanced age, delay in diagnosis and management, extension of gangrene beyond the perineum and septic shock. In summary, despite the better understanding of its etiopathogenesis, the advent of targeted antibiotic therapy, the establishment of a better codification of surgical procedures, the contribution of hyperbaric oxygenation and reconstruction techniques, mortality rates are still high and FG remains a real health threat, thus constituting a real medical and surgical emergency.

### What is known about this topic

- FG is a serious infectious disease that is often diagnosed late;
- Its diagnosis is primarily clinical;
- It represents a real medical and surgical emergency; aggressive surgical debridement remains to be the cornerstone of therapy and is commonly preceded by patient preparation for the surgical.

### What this study adds

- Helping to more understand this pathology by explaining its physiopathology;
- Explaining the traditional treatment;
- Insisting on the new means of treatments such as hyperbaric oxygen therapy (HBOT) and the VAC (vaccum assisted closure), and Healing techniques once the septic risk is controlled.
